# Comparative pathogenesis of type 1 (European genotype) and type 2 (North American genotype) porcine reproductive and respiratory syndrome virus in infected boar

**DOI:** 10.1186/1743-422X-10-156

**Published:** 2013-05-21

**Authors:** Kiwon Han, Hwi Won Seo, Changhoon Park, Yeonsu Oh, Ikjae Kang, Chanhee Chae

**Affiliations:** 1 Department of Veterinary Pathology, College of Veterinary Medicine, Seoul National University, 599 Gwanak-ro, Gwanak-gu, Seoul, 151-742, Republic of Korea

**Keywords:** Boar, Genotype, Male reproductive system, Porcine reproductive and respiratory syndrome virus, Semen

## Abstract

**Background:**

Porcine reproductive and respiratory syndrome virus (PRRSV) now has two main genotypes, genotype 1 (European) and genotype 2 (North American). There is a lack of data on the comparison of pathogenicity of the two genotypes in boars. The objectives of the present study were to evaluate the amount of PRRSV present in semen over time and compare the viral distribution and microscopic lesions of type 1 and type 2 PRRSV-infected boars.

**Methods:**

Twenty-four 8-month-old PRRSV-naïve Duroc boars were randomly allocated to 3 treatment groups. The boars in groups 1 (*n* = 9) and 2 (*n* = 9) were intranasally inoculated with type 1 or type 2 PRRSV, respectively. The boars in groups 1 (*n* = 6) served as negative controls. Semen and blood samples were collected up to 35 days post-inoculation (dpi), and necropsies were performed on 14, 21, and 35 dpi.

**Results:**

There were no significant differences in the genomic copy number of PRRSV, microscopic testicular lesion score, number of PRRSV-positive germ cells, or number of apoptotic cells between the type 1 and type 2 PRRSV-infected boars throughout the experiment. Histopathological changes were manifested by the desquamation of spermatocytes and the presence of multinucleated giant cells in seminiferous tubules of both type 1 and type 2 PRRSV-infected boars. The distribution of PRRSV-positive cells was focal; the virus was found in single germ cells or small clusters of germ cells, localized to the spermatogonia, spermatocytes, spermatids, and non-sperm cells in type 1 and type 2 PRRSV-infected boars.

**Conclusions:**

The results of this study demonstrated that two genotypes of PRRSV do not have significantly different virulence toward the male reproductive system of pigs.

## Background

Porcine reproductive and respiratory syndrome virus (PRRSV) is an enveloped arterivirus discovered in late 1980s, with a positive-sense single-stranded RNA genome of approximately 15 kb [1]. PRRSV occurs in 2 major clinical forms: reproductive failure in sows and respiratory disease in growing pigs [2]. PRRSV now has two main genotypes; genotype 1 (European) and genotype 2 (North American) [3]. These genotypes cause similar clinical signs but differ significantly virulence. Type 2 PRRSV induces more severe respiratory disease than type 1 PRRSV, although early outbreaks of PRRS in Europe often arose from type 1 PRRSV infection in sows [4-6].

PRRSV affects boars at all ages and produces various clinical signs such as anorexia, fever, lethargy, and loss of libido [7-10]. Although the pathogenesis of reproductive disease caused by type 1 and type 2 PRRSV in boars has been studied [8,10], there is a lack of data on the comparative pathogenicity of the two genotypes in boars. Hence, the objective of this study was to compare type 1 and 2 PRRSV-infected boars based on viremia, seminal shedding, sites of viral replication, degree of apoptosis, and histopathological lesions.

## Results

### Clinical signs

The daily rectal temperatures of the infected boars increased (39.3-39.5°C) between 3 and 5 days post-inoculation (dpi). Some infected boars were depressed and anorectic for approximately 4 to 7 dpi. After the onset of fever, clinical signs of disease were not observed in the infected boars. The negative control boars had clinically normal health and rectal temperature (38–39.1°C) throughout the experiment.

### Serology of PRRSV

Anti-PRRSV IgG antibodies were detected in infected boars as early as 7 dpi and all the infected boars were found to be seropositive by 10 dpi. Thereafter, all of the infected boars remained seropositive for PRRSV. There was no significant difference in the S/P ratio of the serum samples between type 1 and type 2 PRRSV-infected boars throughout the experiment (p 1). No anti-PRRSV IgG antibodies were detected in the serum of the negative control boars throughout the experiment.

**Figure 1:**
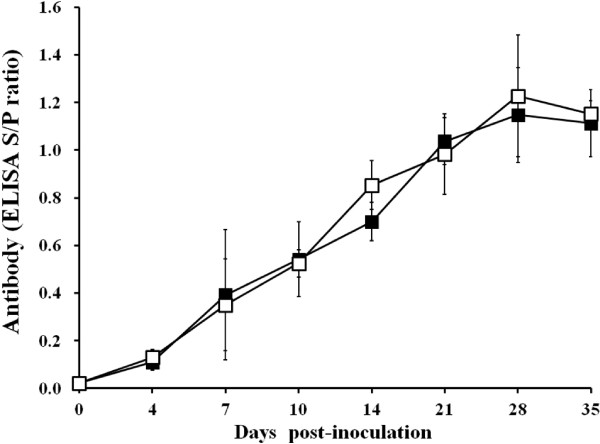
Antibody responses of boars experimentally infected with type 1 and type 2 porcine reproductive and respiratory syndrome virus by the enzyme-linked immunosorbent assay (ELISA; type 1 ■; type 2 □).

### Virus isolation and sequence analysis in blood and semen

Type 1 and type 2 PRRSV were isolated from the testicular tissues of all the infected boars except for two type 1 PRRSV-infected boars at 35 dpi and one type 2 PRRSV-infected boar at 35 dpi. All type 1 and type 2 PRRSV isolated from the infected boars was confirmed by sequence analysis to be the same propagating virus as the challenge stock. No PRRSV was isolated from the testicular tissues in the negative control boars.

### Quantification of PRRSV RNA in blood and semen

**Figure 2:**
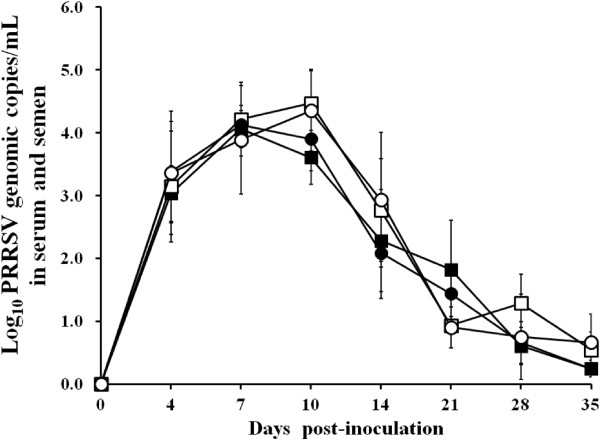
Mean values of the genomic copy numbers of porcine reproductive and respiratory syndrome virus (PRRSV) cDNA in serum (type 1 PRRSV, ■ and type 2 PRRSV, ●) and semen (type 1 PRRSV, □ and type 2 PRRSV, ○) samples from experimentally infected boars.

No genomic copies of type 1 or type 2 PRRSV were detected in semen or serum samples from any of the boars at 0 dpi in 3 groups. Genomic copies of type 1 PRRSV were detected in the serum and semen samples from type 1 PRRSV-infected boars. For the within-group comparison, the number of genomic copies of type 1 PRRSV in sera increased from 0 to 7 (*P* < 0.05) dpi but decreased thereafter. The number of genomic copies of type 1 PRRSV in semen increased from 0 to 7 (*P* < 0.05) dpi but decreased from 10 to 21 dpi. Genomic copies of the type 2 PRRSV were detected in the serum and semen samples from type 2 PRRSV-infected boars only. The number of genomic copies of type 2 PRRSV in serum increased from 0 to 4 (*P* < 0.05) dpi but decreased from 10 to 14 dpi and from 21 and 28 dpi. The number of genomic copies of type 2 PRRSV in semen increased from 0 to 7 (*P* < 0.05) dpi but decreased from 14 to 21 dpi (Figure 2).

For the inter-group comparison, no significant difference in the number of genomic copies of PRRSV was observed in serum and semen samples between type 1 and type 2 PRRSV-infected boars throughout the experiment. No genomic copies of the type 1 and type 2 PRRSV were found in the serum or semen samples from the negative control boars throughout the course of the experiment.

### Microscopic lesions

There was no significant difference in the testicular lesion scores of the type 1 and type 2 PRRSV-infected boars (Table 1). Microscopic lesions were observed in PRRSV-infected boars but not in the negative control boars. Regardless of the type of PRRSV infection, a consistently observed lesion was hypospermatogenesis, which was characterized by a complete lack of mature spermatids and a nearly complete absence of germ cells. The histopathological changes were manifested by the desquamation of spermatocytes and the presence of multinucleated giant cells in the seminiferous tubules of both the type 1 and type 2 PRRSV-infected boars.

**Table 1:** Testicular lesion score (TLS), in situ hybridization (ISH), and apoptosis in boars at different days post-inoculation (dpi)

**DPI**	**n**	**PRRSV**	**TLS**	**Number of positive germ cells for ISH**				**Apoptosis**
				**Spermatogonia**	**Spermatocyte**	**Spermatid**	**Non-sperm cell**	
14	3	Type 1	5.9 ± 1.5	1.4 ± 0.8	2.5 ± 0.6	2.0 ± 0.6	1.4 ± 0.4	35.2 ± 1.4^b^
		Type 2	6.0 ± 1.4	1.1 ± 0.6	2.1 ± 0.4^a^	1.9 ± 0.7	1.4 ± 0.4	28.4 ± 5.8
21	3	Type 1	7.1 ± 1.0	0.9 ± 0.5	1.9 ± 0.5^a^	1.1 ± 0.7	1.2 ± 0.3	23.6 ± 7.2
		Type 2	7.2 ± 1.2	0.9 ± 0.5	1.7 ± 0.4	1.3 ± 0.4	1.1 ± 0.7	21.6 ± 6.8
35	3	Type 1	8.5 ± 0.5	0.5 ± 0.7	0.9 ± 0.5	0.5 ± 0.5	0.3 ± 0.4	16.0 ± 4.2
		Type 2	8.4 ± 0.6	0.4 ± 0.6	0.8 ± 0.4	0.6 ± 0.4	0.4 ± 0.5	16.2 ± 6.4

^a^Significantly different PRRSV-positive cells between spermatogonia and spermatocyte.

^b^Significantly different numbers of apoptosis between type 1 and type 2 PRRSV-infected boars.

### In situ hybridization (ISH)

**Figure 3:**
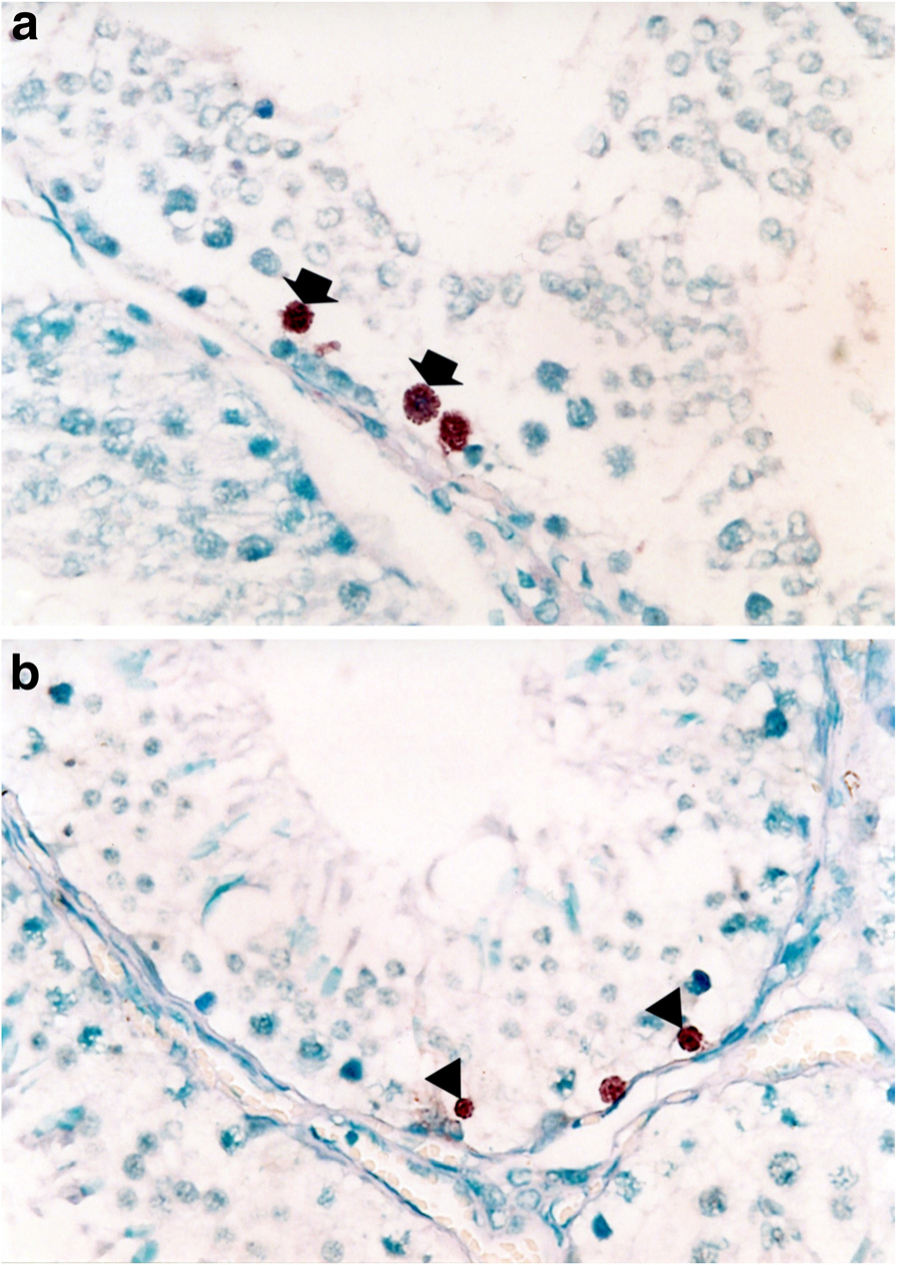
**In situ hybridization of testicular tissues from boars experimentally infected with porcine reproductive and respiratory syndrome virus (PRRSV) at 14 days post-inoculation.** Type 1 (**a**) and type 2 (**b**) PRRSV nucleic acids are detected in spermatocyte (arrow) and spermatogonia (arrowheads).

Type 1 and type 2 PRRSV-positive cells were found in the testes of the type 1 and type 2 PRRSV-infected boars, respectively. No hybridization signal was observed in tissue sections pretreated with RNase A. The distribution of positive cells was focal, in single or small clusters of germ cells with the virus localized to the spermatogonia, spermatocytes, spermatids, and non-sperm cells in both type 1 (Figure 3a) and type 2 (Figure 3b) PRRSV-infected boars.

In the within-group comparison, the number of type 1 PRRSV-positive cells was significantly (*P* = 0.050) higher in spermatocytes than in other cell types in infected boars at 21 dpi. The number of type 2 PRRSV-positive cells was significantly (*P* = 0.048) higher in spermatocytes than in other cell types in infected boars at 14 dpi (Table 1). In the inter-group comparison, there was no significant difference in the number of positive cells among the spermatogonia, spermatocytes, spermatids, and non-sperm cells of type 1 and type 2 PRRSV-infected boars throughout the experiment (Table 1).

Hybridization signals were also detected in epididymal tissues from infected boars. Most of the infected cells were in the lumen of the efferent ducts. The non-sperm cells contained viral nucleic acid. Occasionally, positive cells were also observed in the stromal connective tissue in the epididymis, ductus deferens, and prostate gland. The spermatozoa were consistently negative by ISH for type 1 and type 2 PRRSV in the lumen of the ductus deferens. No hybridization signals were detected in the bulbourethral glands or penis of infected boars.

Positive hybridization signals were not detected in tissues from the negative control boars. Positive hybridization signals were not detected in the testes or epididymis of boars infected experimentally with type 1 PRRSV using the type 2-based PRRSV probe and vice versa. Positive hybridization signals were detected in the lungs from pigs infected experimentally with type 1 (or type 2) PRRSV using only the type 1 (or type 2)-based PRRSV probe, respectively.

### Immunohistochemistry (IHC) of seminal ejaculates

**Figure 4:**
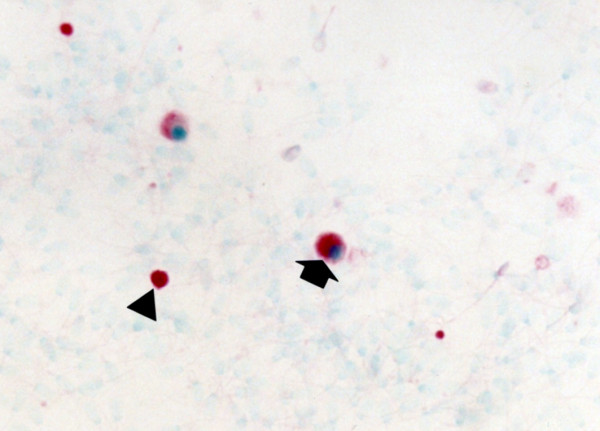
**Immunohistochemistry of semen smear from boars experimentally infected with type 2 porcine reproductive and respiratory syndrome virus (PRRSV) at 14 days post-inoculation.** PRRSV antigen is detected in the non-sperm cells (arrow) and spermatocyte (arrowhead).

PRRSV-positive cells were detected as early as 4 dpi in the ejaculates of type 1 and type 2 PRRSV-infected boars from which semen was obtained. Distinct red staining was observed in the semen smears prepared from samples taken from PRRSV-infected boars. Positive staining of spermatogonia, spermatocytes, spermatids, and non-sperm cells (Figure 4) in the ejaculates was most frequently noted between 7 and 14 dpi.

**Figure 5:**
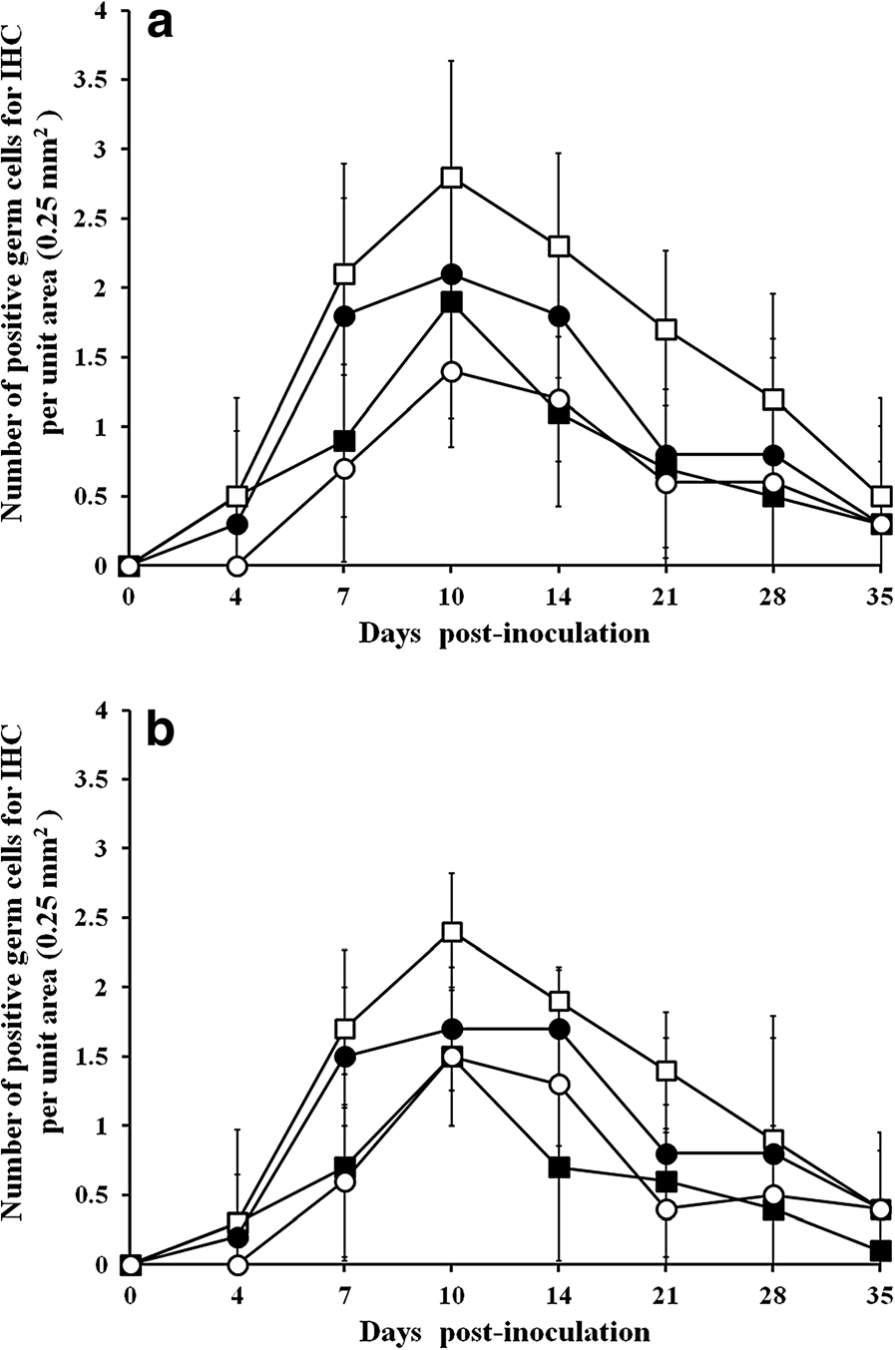
Mean score for immunohistochemistry of semen smear (spermatogonia, ■; spermatocytes, □; spermatids, ●; and non-sperm cells, ○) in pigs experimentally infected with type 1 (a) and type 2 (b) porcine reproductive and respiratory syndrome virus.

In the within-group comparison, the spermatocytes had significantly higher numbers of PRRSV-positive cells than did the non-sperm cells (*P* = 0.034) at 10 dpi, and the spermatogonia (*P* = 0.025) and non-sperm cells (*P* = 0.043) at 14 dpi in type 1 PRRSV-infected boars (Figure 5a). In type 2 PRRSV-infected boars, the spermatocytes had significantly higher numbers of PRRSV-positive cells than non-sperm cells at 7 (*P* = 0.037) dpi, and spermatogonia at 10 (*P* = 0.035) and 14 (*P* = 0.005) dpi. Spermatids had significantly higher numbers of PRRSV-positive cells than did spermatogonia at 14 dpi (Figure 5b). In the inter-group comparison, there was no significant difference in the number of positive cells among the spermatogonia, spermatocytes, spermatids, and non-sperm cells between type 1 and type 2 PRRSV-infected boars throughout the experiment. No PRRSV-positive-stained cells were observed in semen smears prepared from the negative control boars.

### In situ TUNEL staining

**Figure 6:**
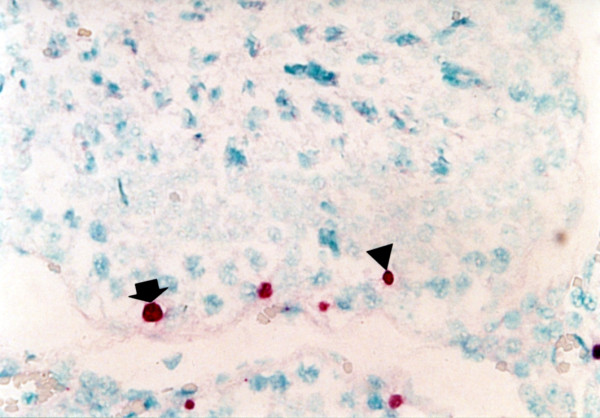
Apoptotic signals are detected in spermatocytes (arrow) and spermatogonia (arrowhead) in boras experimentally infected with type 1 porcine reproductive and respiratory syndrome virus at 14 days post-inoculation.

Apoptosis induced by type 1 and type 2 PRRSV was examined in the testes of type 1 (Figure 6) and type 2 PRRSV-infected boars. TUNEL-positive cells had red-stained nuclei. Examination of serial sections revealed that the PRRSV-positive cells did not co-localize with apoptotic cells. Minimal numbers of apoptotic cells were observed in testicular tissues from negative control boars (4.7 ± 2.1 cells/0.25 mm^2^ for boar at 14 dpi, 4.2 ± 2.9 cells/0.25 mm^2^ for boar at 21 dpi and 5.1 ± 1.3 cells/0.25 mm^2^ for boar at 35 dpi).

In the within-group comparison, the number of TUNEL-positive cells decreased significantly in the type 1 PRRSV-infected boars from 14 to 21 (*P* = 0.019) dpi and from 21 to 35 (*P* = 0.031) dpi. The number of TUNEL-positive cells decreased significantly (*P* = 0.050) in the type 2 PRRSV-infected boars from 21 to 35 dpi (Table 1). In the inter-group comparison, the number of TUNEL-positive cells at 14 dpi were significantly (*P* = 0.035) higher in testicular tissues of the type 1 PRRSV-infected boars than in those of the type 2 PRRSV-infected boars (Table 1).

## Discussion

This study demonstrated that type 1 and type 2 PRRSV were able to infect germ cells of male reproductive organs from infected boars. Infection of PRRSV may be depended on the presence of viral receptors on the surface of germ cells. PRRSV enters porcine alveolar macrophages via the receptor-mediated endocytosis in vivo [11]. Four receptors of PRRSV have been identified on porcine macrophages: heparan sulphate, sialoadhesin (CD169), CD 163, and CD151 [12-14]. Further studies are needed to determine the presence of these receptors or other possible receptors on germ cells of male reproductive organs.

The simultaneous detection of viral nucleic acid and protein of type 1 PRRSV by ISH and IHC, respectively, in spermatogonia and other cells indicates that PRRSV replicates in these cells. No significant differences in the type 1 and type 2 PRRSV-positive cells in the male reproductive organs were observed in the present study. These observations contrast with those regarding the respiratory disease in growing pigs [4,5,15], in which type 2 PRRSV was found in higher titers in the respiratory organs, especially alveolar macrophages. These results suggest that type 2 PRRSV may have more affinity for the macrophage lineages. However, there is no significant difference in the affinity of type 1 and type 2 PRRSV for cells of non-macrophage lineages, such as germ cells of male reproductive organs.

This study demonstrated that PRRSV were localized prominently in spermatogonia and their progeny, and apoptosis were also seen in these germinal cells. These results agree with previous findings in which the major site of PRRSV replication and apoptosis were germinal cells; spematogonia, spematocytes, and spermatids [10]. Spermatogonia are one of the most important potential targets because these cells do not constitutively express biologically active interferon [16]. PRRSV was most consistently found in the cell fractions of semen from vasectomized and nonvasectomized boars [17,18]. These observations suggest that PRRSV excretion in semen results from viral replication in the reproductive tract of the boars or originates from other organs or tissues in a cell-free or cell-associated state. Our results were also shown that germinal cells are the major contributors of cell-associated PRRSV in ejaculates.

The origin of PRRSV in spermatogonia and their progeny was most likely to the hematogenous spread of the virus because PRRSV did not infect the epithelial cells lining the ducts or the glands of the male reproductive tracts. PRRSV antigens were predominantly detected in macrophages in testes and other male reproductive tracts [18]. PRRSV may use macrophages as a vector for spread of infection to germinal cells within testes. Of the blood vessels, monocytes and endotheial cells have been associated with viremia [19,20]. Because monocytes continue to circulate, virus dissemination in reproductive organs such as testes occurs via infected monocytes. PRRSV viremia contributes to viral distribution throughout the reproductive tissues [18]. Alternatively, direct infection from infectious virions in the peripheral blood is also a distinct possibility given the highly vascular nature of the testes.

There is no significant difference in the histopathological lesions of type 1 and type 2 PRRSV-infected boars. The main histopathological lesions are desquamation and multinucleated giant cells of germ cells in both type 1 and type 2 PRRSV-infected boars. These characteristics lesions were also described in previous study [10]. The observed desquamation of germ cells indicated the disruption of spermatogenesis [10]. The multinucleated giant cells, consisting of degenerated and necrotic speromatocytes and/or spermatids [21] are the consequence of the particular mode of cytokinesis of dividing germinal cells. The failure of cytokinesis is one of the causes of origin of multinucleated giant cells [22].

Analysis of ejaculates by IHC demonstrated that PRRSV protein was present in spermatogonia and their progeny, and non-sperm cells. The origin of non-sperm cells was identified as macrophages [18]. This result is in agreement with previous findings that PRRSV is present in semen mainly in a cell-associated fashion [10,23,24]. Detection of PRRSV-infected spermatogonia and their progeny in ejaculated semen indicated that the virus is certainly released from the seminiferous epithelium [25] and that the semen may contribute to spreading the virus to the sows [26]. Several studies have demonstrated seroconversion of sows and gilts bred with infected boars or inseminated with semen from such boars or with experimentally contaminated semen [7,26-29].

Boar vaccination against the PRRSV infection is important preventive tool because of the prolonged seminal shedding of virus [13,26]. Vaccination with the modified live PRRS vaccine reduced or eliminated shedding of wild-type PRRSV in challenged boars by day 50 following vaccination [30]. Nevertheless, the cross-protection between two genotypes is another issue because type 1 PRRSV emerged in Asian countries [31-33]. It has been reported that the type 2 PRRSV-based modified live vaccine is more effective against type 2 PRRSV than the type 1 PRRSV in vaccinated and challenged boars [34].

This is the first study to compare the amount of PRRSV cDNA shed in semen of type 1 and type 2 PRRSV-infected boars. Quantitative differences in the shedding patterns of the two viral genotypes were not observed under the experimental conditions. There was no significantly difference in viral replication or the microscopic testicular lesions associated with the two genotypes. Therefore, two genotypes of PRRSV do not differ significantly in their virulence toward the male reproductive system of pigs. However, our results should be interpreted carefully because the pathogenesis of PRRSV can vary greatly among different strains of the same genotype [4,35]. In addition, it should be pointed out that this experiment used one strain of virus of each genotype, which has been passaged serially in MARC-145 cells. It is important to use inoculate which have undergone minimal passage in a porcine line ideally alveolar macrophages because the pathogenicity of the PRRSV field isolate became attenuated when propagated in MARC-145 cells [36]. Further studies are needed to use several strains of virus in each genotype to compare the pathogenesis in male reproductive organs.

## Conclusions

In this study, the comparative pathogenicity between type 1 and type 2 PRRSV in boars was evaluated. No significant difference in the number of genomic copies of PRRSV, the microscopic testicular lesion score, PRRSV-positive germ cells, and the numbers of apoptotic cells between type 1 and type 2 PRRSV-infected boars throughout the experiment. The results suggest that the two viral genotypes of PRRSV do not differ significantly in their virulence toward the male reproductive system of pigs.

## Materials and methods

### PRRSV inoculum

**Figure 7:**
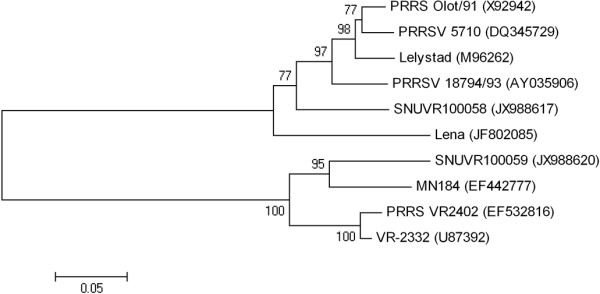
**Phylogenetic analysis of open reading frame 5 of porcine reproductive and respiratory syndrome virus (PRRSV) genome.** An unrooted neighbor-joining tree was constructed from aligned nucleic acid sequences of several PRRSV found in GenBank.

Type 1 PRRSV (SNUVR100058) was isolated from lymph node tissue of an aborted fetus in 2011 in Kyounggi Province. Type 2 PRRSV (SNUVR100059) was isolated from lymph node tissue of an aborted fetus in 2011 in Chungcheung Province. Type 1 and type 2 PRRSV (passage 6) were propagated in MARC-145 cells to a titer of 1 × 10^6^ 50% tissue culture infective dose (TCID_50_)/mL. SNUVR100058 strain was identified as type 1 PRRSV on the basis of nucleotide sequences of open reading frame (ORF) 5 (GenBank JX988617) and ORF 7 (GenBank JX988612). SNUVR100059 strain was identified as a type 2 PRRSV based on the nucleotide sequences of ORF 5 (GenBank JX988620) and ORF 7 (GenBank JX988615). Type 1 and type 2 used in this study share 58.7 and 60.8% nucleotide identity for ORF5 and ORF7, respectively. Type 1 and type 2 PRRSV used in this study were analyzed phylogenetically along with prototype of PRRSV (Lelystad, VR-2332, and Lena) and some 5 other strains used in male reproductive studies that are listed in the GenBank database as ORF 5 sequences (Figure 7).

### Experimental design

At 8 months of age, 24 purebred male Duroc pigs were purchased from a PRRSV-free commercial farm. The boars were PRRSV negative according to routine serology and real-time polymerase chain reaction (PCR) prior to delivery and on arrival. All boars were individually housed throughout the experiment in an environmentally controlled building with pens over completely slatted floors.

The boars were randomly allocated to 3 groups. The boars in group 1 (*n* = 9) were inoculated with type 1 PRRSV intranasally (2 mL) with an infectious titer of 1 × 10^6^ TCID_50_/mL. The boars in group 2 (*n* = 9) were inoculated with type 2 PRRSV intranasally (2 mL) with an infectious titer of 1 × 10^6^ TCID_50_/mL. The boars in group 3 (*n* = 6) served as negative controls. Following PRRSV inoculation, the physical condition of the boar was monitored daily and their rectal temperatures were taken. Each of three infected and two negative control boars were tranquilized by an intravenous injection of azaperon (Stresnil, Janssen Pharmaceutica, Beerse, Belgium) and then euthanized by electrocution for necropsy at 14, 21, and 35 dpi. Tissues were collected from each pig at necropsy. All of the methods were previously approved by the Seoul National University Institutional Animal Care and Use Committee (SNU-121001-4).

### Serology

Blood samples from boars were collected at 0, 4, 7, 10, 14, 21, 28, and 35 dpi. The serum samples were tested using the commercially available PRRSV enzyme-linked immunosorbent assay (ELISA; HerdCheck PRRS 2XR™, IDEXX Laboratories Inc., Westbrook, Maine, USA). Serum samples were considered positive for PRRSV antibody if the S/P ratio was greater than 0.4 according to the manufacturer’s instructions.

### Virus isolation

Testicular tissues from boars were collected for virus isolation from all infected and negative control pigs. PRRSV was isolated from these organs as previously described [37].

### Sequence analysis

PRRSV isolated from testicular tissues was further analyzed for the ORF5 sequence. RNA was extracted from PRRSV-infected MARC-145 cells [37] and amplified from the ORF5 region by reverse transcription (RT)-PCR [38]. Sequencing was performed on the purified RT-PCR products of amplified ORF5.

### Quantification of PRRSV RNA

RNA extractions from the semen (raw) and serum samples were collected 0, 4, 7, 10, 14, 21, 28, and 35 dpi from boars used in this study. Real-time PCR for the type 1 and type 2 PRRSV was used to quantify PRRSV genomic cDNA copy numbers using RNA extraction from semen and serum samples which were performed as previously described [39]. The real-time PCR was considered to be positive if the cycle threshold level was obtained at <45 cycles [39].

### Preparation of labeled probe

For type 1 PRRSV, a 354-base-pair cDNA fragment representing the 5' region of the ORF6 and ORF7 was used as a probe. The forward and reverse primers were 5'-CGCTGTGACAAAGCCCGGAC-3' (nucleotides 14,482 to 14,501) and 5'-TCGATTGCAAGCAGAGGGAG-3' (nucleotides 14,814 to 14,835), respectively. For type 2 PRRSV, a 349-base-pair cDNA fragment representing the 5' region of the ORF6 and ORF7 was used as a probe. The forward and reverse primers were 5'-TCGTTCGGCGTCCCGGCTCC-3' (nucleotides 14,775 to 14,794) and 5'-TTGACGACAGACACAATTGC-3' (nucleotides 15,122 to 15,141), respectively. RT-PCR was carried out as previously described [40]. The purified RT-PCR product was labeled by random priming with digoxigenin-dUTP using a commercial kit (Boehringer Mannheim).

### In situ hybridization (ISH)

Five serial sections (4 μm) were mounted on positively charged slide (Erie Scientific Company, Portsmouth, NH, USA) and then prepared from each tissue, two being further processed for ISH using a type 1 and 2 PRRSV probe without RNase A treatment, two for ISH using a type 1 and 2 PRRSV probe with RNase A treatment and one for haematoxylin and eosin (HE) staining. ISH was performed as previously [41]. The lung tissues from three type 1 and type 2 PRRSV-infected pigs euthanized at 7 dpi were used as positive controls for ISH.

### Immunohistochemistry (IHC)

Whole semen was filtered through a triple layer of gauze and diluted 1:1000 in Eagle's minimum essential medium. Diluted whole semen was applied to positively charged slide (Erie Scientific Company) by centrifugation at 200 ×*g* for 5 min. Slides were fixed in alcohol for 5 min and air dried. IHC for type 1 and type 2 PRRSV was performed using SR30 monoclonal antibody (Rural Technologies Inc., Brookings, SD, USA) as previously described [42]. SR30 monoclonal antibody (Rural Technologies Inc.), capable of specifically recognizing nucleocapsid protein of PRRSV, was diluted 1:10,000 in PBS (0.01 M, pH 7.4) containing 0.1% Tween 20.

### In situ TUNEL staining

Tissue sections (4 μm) were mounted on positively charged slide (Erie Scientific Company), deparaffinized, and rehydrated. Sections and slides were first treated with 20 μg/mL of proteinase K in phosphate buffered slaine (PBS, 0.1 M, pH 7.4) for 20 min at 37°C. After washing in PBS, sections and slides were covered with 50 μl of the TUNEL reaction mixture (Boehringer Mannheim, Indianapolis, IN, USA) and incubate under a coverslip in a humidified chamber for 1 h at 37°C. The reaction was stopped by washing slides in PBS for 15 min at room temperature. The sections were then incubated with the anti-fluorescein-alkaline phosphatase conjugate (Boehringer Mannheim) diluted 1/3 in 100 mM Tris–HCl, 150 mM NaCl (pH 7.5), and 1% blocking agent for 1 h at room temperature. After three washes in PBS, substrate consisting of nitroblue tetrazolium (NBT) and 5-bromocresyl-3-indolylphosphate (BCIP) was layered over the sections. Color was allowed to develop for 5–8 h in the dark, and the reaction was stopped by dipping slides briefly in Tri-ethylenediaminetetraacetic acid buffer (10 mM Tris–HCl and 1 mM EDTA, pH 8.0). Sections were counterstained with 0.5% methyl green, and the slides were then washed with distilled water for 1 min, and then allowed to dry completely.

### Morphometric analysis

Five sections of formalin-fixed testes were taken from each virus-infected boar for morphometric analysis. Morphometric analysis of the microscopic testicular lesions was performed as previously described [43]. In each testicular slide, spermatogenesis was determined by the semiquantitative testicular score count in 100 cross-sections in each boars at the same magnification and was summed up as mean testicular score. Five sections of formalin-fixed testes were taken from each virus-infected boar for morphometric analysis. In each testicular slide, 10 fields were randomly selected, and the number of positive cells by ISH and in situ TUNEL per unit area (0.25 mm^2^) was counted. The seminal ejaculates from PRRSV-infected boars were also used for morphometric analysis. Numbers of positive spermatogonia, spermatocytes, spermatids, and non-sperm cells per unit area (0.25 mm^2^) by IHC were also measured in seminal ejaculates.

### Statistical analysis

Student’s *t*-test for paired samples (testicular lesion score, ISH, in situ TUNEL, and IHC scores) was used to compare the differences between type 1 and type 2 PRRSV-infected boars. Continuous data for PRRSV RNA quantification over time among the groups were analyzed at each time point using Paired *t*-test. *P* < 0.05 indicated statistical significance.

## Competing interests

The authors declare that they have no competing interests related to the present study.

## Authors’ contributions

KH and HWS performance of the experimental trials, data analysis and writing of the manuscript, CP and YO preparation of the inoculum and lab analysis, IK inoculation of virus, CC development of protocol, design of the study, review of the final manuscript, approval for publication. All authors read and approved the final manuscript.
